# *“The more children you have, the more praise you get from the community”:* exploring the role of sociocultural context and perceptions of care on maternal and newborn health among Somali refugees in UNHCR supported camps in Kenya

**DOI:** 10.1186/s13031-019-0195-z

**Published:** 2019-03-29

**Authors:** Stephanie Gee, Josep Vargas, Angel M. Foster

**Affiliations:** 1 United Nations High Commissioner for Refugees, Case Postale 2500, CH-1211 Genève, 2, Dépôt Switzerland; 2 Senior Reproductive Health & HIV Officer, United Nations High Commissioner for Refugees, Case Postale 2500, CH-1211 Genève, 2, Dépôt Switzerland; 3 University of Ottawa, 1 Stewart Street, 312-B, Ottawa, ON K1N6N5 Canada

**Keywords:** Reproductive health, Maternal and newborn health, Refugee camp, Somali, Kenya

## Abstract

**Background:**

Maternal and neonatal survival are key components of population health and may be particularly vulnerable in humanitarian contexts of civil unrest and displacement. Understanding what factors contribute to poor health outcomes throughout the reproductive life cycle and across the continuum of care is crucial for improving health programming in acute and protracted refugee settings.

**Methods:**

We undertook a mixed-methods baseline assessment of factors related to maternal and neonatal health among refugees living in the Dadaab refugee complex in eastern Kenya. The qualitative component included 23 focus group discussions with 207 community members and 22 key informant interviews with relevant UN and non-governmental organization staff, community leaders, health managers, and front-line health care providers. We analysed qualitative data for content and themes using inductive and deductive techniques.

**Results:**

Taking a life course perspective, we found that the strong desire for large families and the primary social role of the woman as child bearer impacted maternal and neonatal health in the camps through preferences for early marriage, low demand for contraception, and avoidance of caesarean sections. Participants described how a strong fear of death, disability, and reduced fecundity from caesarean sections results in avoidance of the surgery, late presentation to the health facility in labour, and difficulty gaining timely informed consent. Mistrust of health service providers also played a role in this dynamic. In terms of newborn care practices, while breastfeeding is culturally supported and women increasingly accept feeding colostrum to the newborn, mixed feeding practices and application of foreign substances to the umbilicus continue to present risks to newborn health in this community.

**Conclusions:**

The findings from our study showcase the role that specific sociocultural beliefs and practices and perceptions of health care services have on maternal and neonatal health. An in-depth understanding of how these factors impact the utilization of biomedical health services provides valuable information for targeted improvements in health service provision that are tailored to the local context.

## Introduction

Despite improving global trends, maternal and neonatal mortality rates remain unacceptably high, particularly for women and children living in fragile states and humanitarian settings [1]. Women and children fleeing conflict face numerous threats to their health during displacement including the breakdown of health systems and social supports, food insecurity, and exposure to violence, including gender based violence [2–4]. Access to, and utilization of, key health services in refugee settings, such as family planning, antenatal and postnatal care, skilled birth attendance, and emergency obstetric and neonatal care are essential, life-saving services that can help mitigate vulnerabilities [5]. However, previous service reviews in post-emergency refugee settings have found that despite their availability, the utilization rates of many key reproductive health services often fall below target, suggesting further efforts are required to identify and reduce potential barriers to care [6, 7].

Beyond health services, maternal and neonatal outcomes in refugee settings are influenced by a multiplicity of sociocultural and contextual determinants across the life course – from preconception, through pregnancy, childbirth and into the postnatal period. Gender inequality, low education levels, poverty, and malnutrition, which are common in refugee settings [8], are known to contribute to poor maternal and neonatal outcomes [9]. Early marriage, which may increase as a coping mechanism during displacement [10, 11], increases numerous health risks for both the mother and her newborn if it results in adolescent pregnancy [5]. Sociocultural norms that tend towards a large ideal family size and low contraceptive prevalence rates may similarly increase risks of both maternal and neonatal mortality [12]. Finally, the local sociocultural and contextual factors that influence home-based care practices and care-seeking behaviors, such as decisions to breastfeed or utilize skilled birth attendance, are crucial for health service providers to understand and to take into consideration.

Given the complex challenges faced by women of reproductive age and their newborns in refugee settings, focused attention is required to improve health outcomes. With the aim of improving newborn health outcomes, UNHCR implemented the project “Saving Newborn Lives in Refugee Settings” from 2016 to 2018 in refugee camps in Jordan, South Sudan, and Kenya with the support of the Bill and Melinda Gates Foundation. In order to better understand the local context and priorities for intervention we carried out a mixed methods baseline assessment in 2016. The assessment comprised three components: 1) a document and literature review, including a scoping review of the anthropological literature; 2) quantitative health facilities assessments; and 3) a qualitative exploration of the knowledge, beliefs, and practices of refugees as they relate to maternal and newborn health. This paper presents qualitative findings related to the influence of sociocultural context and perceptions of care on maternal and newborn health in the Dadaab refugee camps of Eastern Kenya.

### The Dadaab context

Since 1991, Somalis have fled their country in successive waves, fleeing civil conflict, drought, and related famines. In 2017 the Dadaab Refugee Complex in Eastern Kenya, was comprised of 5 separate camps hosting 239,549 refugees, with Somalis making up 96% of the refugee population [13]. Dadaab camp residents come mainly from a nomadic pastoralist background, but also include farmers from areas along the Southern Juba River valley, former civil servants, and traders. The Darod clan and its numerous sub-clans are the most prominent in the camps (UNHCR: Refugee operation update Sub-office Dadaab, Sept 2011, unpublished) Given the almost 25 year-long history of the camps there are now around 10,000 third-generation refugees who have been born in Dadaab, as well as a continuous stream of new arrivals [14].

Somali refugees arrive from a context of dismal reproductive health indicators, including a maternal mortality ratio of 732 per 100,000 live births and a neonatal mortality rate estimated at 38.8 per 1000 live births [15]. In Somalia childbearing begins early and the country has the second highest total fertility rate in the world, at 6.4 children per woman [16]. Less than 10% of births take place with a skilled birth attendant [17]. A significantly better reproductive health context is found in Dadaab refugee camps, where facility-based births accounted for between 61 and 87% of all births in the camps in 2015. However, neonatal deaths were found to be the largest contributor to both all-age and children under five mortality in Dadaab in the same year  (UNHCR: Health Information System Dadaab annual report 2015, unpublished). Death audits found asphyxia, prematurity, and sepsis to be the leading direct causes of neonatal mortality, and contributing factors included late presentation to the health facility during labour; refusal to consent for caesarean section; lack of transportation; and lack of equipment or expertise in the camp health facilities (UNHCR: Neonatal mortality review Dadaab 2015, unpublished). 

Health care services in Dadaab camps are overseen by UNHCR and provided free-of-charge through a range of non-governmental organization (NGO) partners that operate 16 health posts, four hospitals, and one stand-alone maternity centre, in addition to community outreach services. Providing health and other services in the camps is constrained for a number of reasons. Security issues, including kidnappings of humanitarian aid staff, improvised explosive devices, and attacks on refugee leaders and Kenyan police, has heightened security restrictions and makes aid provision challenging.

## Methods

### Study design

The qualitative arm of the baseline assessment took a phenomenological approach, seeking to understand the lived experiences of refugee populations specific to their beliefs and practices around reproductive, maternal and newborn health. We chose to conduct focus group discussions (FGDs) and in-depth interviews due to their ability to provide an in-depth understanding of the social dynamics around reproductive health decisions.

### Data collection

Data collection occurred in February and March 2016 in all five camps of the Dadaab refugee complex. SG and JV, both medical professionals with years of experience working in refugee settings, supervised data collection. We trained teams of facilitators over 3 days to conduct the FGDs, with each team consisting of one facilitator and two note takers. Facilitators were refugees themselves and able to speak and write both Somali and English, with many having past experience with qualitative data collection through leading nutrition-related focus groups. Trained research assistants, who completed some of the in-depth interviews at the health facilities, were professional nurses or midwives working in the camps. The study team took care to ensure they did not interview members of their own organization or health facility.

#### Sampling

We recruited FGD participants using purposive sampling, as we aimed to include a variety of demographic groups in order to get a holistic overview of community perceptions, including those of mothers and fathers with a child under two years of age (to ensure recent experience with camp health services); grandmothers; traditional birth attendants; and community health workers. Local NGO partners working in each camp recruited participants by word of mouth. Participants were not remunerated but refreshments were provided.

Sampling for key informant interviews used both purposive and convenience approaches. We used purposive sampling to identify key informants in leadership roles, including relevant UN and NGO staff, Ministry of Health representatives, community leaders, and health program managers. We then used convenience sampling to recruit front-line health care providers, inviting staff working on the day of the health facility assessment to participate in in-depth interviews.

#### Focus group discussions

We held 23 FGDs in all five camps with a total of 207 refugee participants. To ensure some degree of homogeneity, we structured groups around sex, family position, and occupation and held separate discussions with mothers, fathers, grandmothers, traditional birth attendants, and community health workers (CHWs) (Table 1). Each group consisted of 8–12 participants and lasted an average of 1.5 h. Our discussion guide focused on the key determinants of maternal and newborn health across the reproductive life cycle (preconception, antenatal, childbirth, and postnatal stages) and, together with focus group discussion facilitators, we adapted the questions prior to the sessions and agreed upon culturally relevant language and expressions. We conducted the FGDs in the Somali language and note takers simultaneously translated the discussion into English-language notes. At the end of each session, the facilitators compiled notes and, together with SG or JV, reviewed and agreed on the key findings.

**Table 1:** Focus group participants

Focus group participants	Number of focus groups held	Number of participants
Mothers	7	57
Fathers	4	44
Grandmothers	4	34
TBAs	4	34
CHWs	4	38
Totals	23	207

#### Key informant interviews

We conducted 22 semi-structured in-person interviews over the course of the study (Table 2). SG and JV conducted the majority of key informant interviews in English and face-to-face in the camps; trained research assistants conducted the remainder during the health facility assessments. We received written informed consent and conducted the interviews in private offices within the health facilities. The topic guide covered questions on coordination, referral processes, challenges, strengths, and needs related to service provision, health priorities, and experiences of providing care, among others. Interviewers either audio-recorded the interviews or took extensive notes. We also recorded numerous informal discussions and observations throughout the assessment process in a field journal.

**Table 2:** Key informant interviews

Type of participant	Number of interviews
Health program manager	9
Front-line health worker	9
Ministry of Health official	1
Community leader	2
UNHCR official	1
Total	22

### Data analysis

Our analytic approach included both inductive and deductive techniques [18]. SG transcribed the notes from the FGDs and interviews and read them and the field journal repeatedly to familiarize herself with the data. We then reviewed the data line-by-line and systematically coded it for content [19]; we developed a codebook of both a priori codes based on our review of the literature and the research questions and inductive codes that emerged from the data. After we coded the notes from both study components, we combined our data and used the reproductive life cycle and our domains of inquiry as the scaffolding by which to organize themes. We then explored the relationship between themes and looked for concordant and discordant findings between the individual FGDs and between the FGDs and the key informant interviews. We used the software NVIVO 10 to assist the process of coding and theme organization.

In the results section, we group our findings by themes following the reproductive life cycle, including preconception, pregnancy, childbirth, and the postnatal period. We have redacted or masked all personally identifying information about both FGD participants and key informants.

## Results

### Preconception

#### Family dynamics and social status

According to key informants, early marriage (before 18 years of age) and childbearing continue to be common practice among the Somali populations in the camps. When we asked mothers’ and fathers’ groups about the appropriate age to get married and start childbearing, responses ranged from 9 years to 25 years; with many respondents stating an age below 18. Participants regularly reported that the onset of menstruation is an indicator that a woman is ready for marriage and childbearing. As one participant in the fathers’ FGD explained, even the age of nine was acceptable for marriage if the girl has reached puberty, “Our religion allows it if she is physically mature with breasts and menses at this age”.

The ability to bear a large number of children was felt to be a key advantage of early marriage, and participants in all groups viewed large families as desirable. The benefits conferred on the family (such as extra ration cards and more food) as well as the extra income, household assistance, and caregiving (particularly to older parents) that children provide were cited as the key reasons for desiring large families. Grandmothers’ and mothers’ groups also discussed that women gain increased honour and higher social status by bearing many children, which in turn increases their clan’s power and gives the family a good reputation or “name” in the community. One of the participants in the FGD with mothers offered, “The more children you have, the more praise you get from the community.”

Early marriage also avoids potential scandal from pre-marital relationships among adolescents. One mother explained, “In our culture at 15 years old it is allowed to get married and I support that because the ladies of today are misbehaving and they run after men. So to avoid that, better to marry early.”

At the same time, there was the recognition that early marriage carries health, economic, and social risks, particularly from the mothers’ groups. Mothers referred to the dangers that adolescents experience during pregnancy and childbirth (including giving specific examples of obstructed labour); the inability of married girls to finish school; young girls’ immaturity in taking care of a household; and their susceptibility to the emotional pressures of dealing with the husband and the in-laws.

#### Fears of contraception

Participants in the mothers’ and fathers’ groups indicated that community members may view the use of contraception as interfering with the will of God. Consequently, women do not speak of family planning openly. Given that small families are considered shameful and contraceptive use is stigmatized, family planning providers indicated in key informant interviews that women are hesitant to discuss contraception around other Somalis and at times will seek out non-Somali health staff privately to request a contraceptive method. At the same time, health care workers reported that they often avoid raising the subject of contraception with women due to these sentiments in the community. Mothers reported fear of contraceptive side effects as reasons for non-use, including becoming infertile, bleeding, having headaches, and missing periods. As one young woman stated, “One might end up being barren if injected or used drugs for birth control. You might be cursed and won’t get children in the future.”

#### Advantages of birth spacing

Although participants had generally negative attitudes toward modern contraception, both fathers and mothers in FGDs identified numerous advantages of birth spacing and repeatedly stated that breastfeeding was a “natural” and socially acceptable method of delaying a subsequent pregnancy. Stated benefits of birth spacing included improving the health of the child and the mother, allowing a longer time for breastfeeding, and giving the woman more time for herself.

### Pregnancy and childbirth

#### Self-care in pregnancy

Mothers, fathers and grandmothers in our FGDs recognized pregnancy as a vulnerable state and frequently raised good nutrition as a means of “self care”. FGD participants described eating vegetables and taking iron and vitamin supplements as important. Health worker key informants had a different perception, stating many women thought poorly of oral medications, including iron/folic acid tablets, and often did not take them as prescribed. Mothers’, fathers’ and community health workers’ groups strongly complained about the removal of the “fresh food voucher” program for pregnant women and expressed concern that the quantity and quality of the food ration was insufficient. Mothers’ group participants further explained that the “porridge” offered at the antenatal clinic as part of the supplementary feeding program served as a major incentive for them to participate in antenatal care. However, long wait times and challenges in securing transportation to the health post constituted major barriers to their access to antenatal care services.

### Childbirth

#### Preferred childbirth experiences

Many Somali woman previously delivered at home with the help of a TBA, however, according to key informants, this is largely shifting towards health facility birth following years of targeted health promotion efforts in the camps. With some exceptions, the general consensus among participants in all FGDs was that the first choice of place of delivery is now the hospital. The main reasons for this preference included skilled medical staff, free care, safety, and the receipt a birth notification that eases the process of getting an additional ration card, a highly valued and necessary commodity among the camp populations.

At the same time, mothers’ and fathers’ groups reported numerous barriers to facility delivery which led them to deliver at home, with the delay in the arrival of the ambulance (“Mama taxi”) cited as the most frequent reason given for delivering outside of the health facility. Other barriers, according to mothers’ groups, included negative perceptions of hospital care, such as a strong fear of caesarean section; the inability of family members to help during in labour and delivery; being spoken to rudely or being neglected by the midwife; not being understood by the midwife due to language barriers; and fear of being examined or cared for by male staff. According to key informants, the majority of health care workers in Dadaab camps are relocated Kenyans coming from a different religious, language and cultural background to the Somali population. Maternity staff in all health facilities report that women are not allowed to keep companions with them during labour and birth, and may do so only on the postnatal ward. TBAs and family members who accompany labouring woman to the health facility unhappily described being “chased away at the gate” and being unable to remain to provide supportive care. Highlighting the value of birth companions, one mother explained her reasons for having a home birth, “My people are around me to help me when I am in labour. My relatives are around me to give food and drink. I am around my children instead of being in the hospital.”

#### Fear of caesarean section

According to health care providers in key informant interviews, although facility delivery rates are increasing, *timely presentation* – that is when a woman presents early enough in labour to prevent or manage complications before they become life threatening for mother or baby - remains a serious problem. According to health care workers and program managers the leading reason that women present to health facilities late in labour is due to a fear of caesarean section and women may refuse the procedure when medically indicated. These behaviours contribute to the low caesarean section rate in the camps despite good availability; consequently asphyxia is the leading cause of neonatal mortality in the camps.

Many mothers and grandmothers in the FGDs expressed the belief that a caesarean section will leave them permanently disabled and unable to complete the daily tasks of household life. In addition, there appears to be common fears that death might occur from a spontaneous re-opening of the scar, and that the procedure will limit future reproductive capacity. Some women went on to explain their beliefs that caesareans are done for the benefit of hospital staff, as a way to earn extra money or “remove extra organs for sale”. Finally, mothers also expressed a belief that clinicians give drugs to purposefully delay the delivery so that a caesarean can be performed.

Mothers’ focus groups and community leader key informants revealed how women may face severe social repercussions if they agree to the surgery, including divorce or re-negotiation of the dowry (bride price, paid from the husband’s family to the wife’s). Due to these consequences, social stigma has developed around what are described as “operated” women; these women are considered weak or damaged and thus one must be careful about how one behaves around them. As one FGD participant in the group of mothers offered, “They say ‘Don’t argue with her, she’s been operated.’”

Health care providers and management key informants confirmed the prevalence of these beliefs and reported that it was often difficult to gain timely informed consent for emergency caesarean sections, as clinicians must first consult multiple family and clan members. Although the husband’s consent is important, clan elders are often the main decision makers in these cases. Indeed, the decision-making process may reach high up the clan hierarchy and require the input of numerous members, both locally and those reached by phone in Somalia, creating critical delays.

### Postnatal

#### Mixed feeding practices

Mothers in our FGDs expressed a range of experiences and perspectives on infant feeding practices. All women agreed that breastfeeding should start within the first hour of life but at the same time some viewed the baby as “thirsty” and in need of water or other fluids in addition to breast milk. Mothers and grandmothers in our FGDs reported that it used to be common practice to throw away the colostrum and give the baby sugar water until the first milk came in, but this practice has largely stopped due to health education in the camps. According to midwife key informants, immediately following delivery, women exhibit a state of “exhaustion” and are not expected to perform many activities, including newborn care. Consequently, the mother-in-law will often take over care of the newborn during these first hours of life and the feeding of sugar water or animal milk often begins during this time. Grandmothers also mentioned the practice of giving animal milk mixed with oil shortly after birth to help with the newborn’s digestion. Hospital maternity department staff stated that mothers and mothers-in-law are well informed of recommended practices and will often hide the bottle when they see the nurse approaching. As reported by one professional midwife at Dagahaley Hospital, “Mothers only do the right thing when being supervised but are quick to give bottled water, milk, and sugar water when not being watched*.”*

According to key informants working in the nutrition program, the use of “Anchor”, a brand of powdered skim milk, or infant formula such as “Nan” signals good financial straits and is highly regarded by community members. Fathers play a role in mixed feeding, and men participants expressed that the father ought to have enough money to purchase powdered milk or infant formula; as one father stated, “If you cannot provide powdered milk or formula for your child you are not a good father”.

#### Traditional breastfeeding beliefs and practices

Mothers and grandmothers raised a number of other traditional beliefs and practices around breastfeeding. They explained how women tend to drink large amounts of fluids, particularly tea, in the belief that this will increase milk production. In addition, they stated that many women do not continue breastfeeding when they become pregnant again for fear that the breast milk will poison the baby and cause diarrhoea. Mothers, grandmothers, community health workers and health facility key informants described a belief, that the baby should be fed from the right breast only, as feeding from the left breast will cause the child to stammer when he is older. An alternative version of this belief is that the left breast is “cursed by the forefathers” and feeding a child from this breast will result in its death. One mother reported that two of her infants died due to this curse. Health staff report that significant health education on this topic has resulted in the belief becoming less common, but continues to be heard from new arrivals from Somalia.

#### Newborn umbilical and thermal care

Mothers, grandmothers, and traditional birth attendant participants described how charcoal or charred leaves of the Malmal plant are commonly placed on the newborn’s umbilical stump at home. Both are believed to help dry up the cord and quicken healing. Regarding thermal care, health staff report a strong resistance to early skin-to-skin care due to lack of cooperation from mothers; informal attempts to implement kangaroo mother care had previously been unsuccessful. According to midwives, mothers often keep the newborn at the foot of the bed in the postnatal ward and rarely hold the baby in the hours after birth. They explain this relates to the state of “exhaustion” exhibited by mothers after delivery and the prominent role of the woman’s mother-in-law in providing newborn care in the postnatal ward.

#### Newborn care-seeking behaviour and traditional medicines

Mothers agreed on the importance of having the baby checked at routine postnatal care appointments, but felt discouraged by long lines as well as the inconvenience of leaving the home during the early postnatal period. Mothers stated their first choice for care if their newborn is sick is the health post; if the baby does not get better they will then try traditional herbs or, if they can afford it, go to private pharmacies. Reasons given for choosing the health post include free service, good quality doctors, and safe and reliable care. However, mothers found a key barrier to accessing care with a sick neonate is the lack of transport within the camps as ambulance services are restricted to women in labour. As one mother explained, “When a patient is in critical condition, they fundraise money to help take the patient to the main hospital at night, and if they don’t have the money, the patient dies at home due to lack of transportation.”

Similar to avoidance of interventions during childbirth, hospital staff report difficulty in convincing mothers to allow invasive procedures on the premature or ill newborn, particularly nasogastric feeding tubes and intravenous catheters; if inserted, staff will often find the tubes removed by the mothers. According to health service providers, there is a widespread belief in the community that these interventions are harmful, including a prominent idea that the nasogastric tube is entering the brain.

Elder women, including several grandmothers and TBAs in our study indicated that “Quran therapy” should be the first option when seeking care for a newborn. As one TBA explained, “If the baby becomes sick we can advise her to read the Quran. After that we take her to the health facility*.*” For other elder women, the baby’s condition determined whether or not a traditional healer should be consulted. For example, if the baby was having convulsions or “fainting”, many grandmothers felt the traditional healer was the best choice, whereas if the baby was having difficulty breathing or coughing the clinic was preferable. Further they describe the use of holy water, or *tahlil*, is an important practice with the newborn, as is reading the Quran to the newborn shortly after birth to protect it from harm.

The herb Malmal is widely perceived as having a range of therapeutic properties. In addition to its use on the umbilicus, mothers and grandmothers extolled its value in treating fever (when mixed with water for a bath) and breathing problems (when crushed and placed under the baby’s nose). Other common beliefs expressed by the women include that Malmal also brings about intelligence and a stable personality.

## Discussion

Considering a life course perspective, the findings of this study highlight a multiplicity of factors are influencing health outcomes across the reproductive life cycle. While health services such as family planning, antenatal and postnatal care, skilled birth attendance, and emergency obstetric and neonatal care are key in preventing neonatal and maternal mortalities [20], the availability of these services in Dadaab camps does not ensure utilization. A desire for large families and a strong link between the social status of women and their fecundity was found to contribute to low demand for modern contraceptive methods and avoidance of invasive medical procedures such as caesarean section.

Fears of harm, both social and physical, from caesarean section results in a cohort of women who continue to deliver at home, present to the health facility very late in labour, or refuse the intervention when recommended, increasing risks to both maternal and neonatal health. This is in line with a previous reviews in Dadaab that found delay in the decision to seek care and refusal to seek or accept care as the top two avoidable factors contributing to maternal mortality [21]. Similarly, neonatal mortality audits in Dadaab in 2015 found the main contributing factors to neonatal deaths included late presentation to the health facility during labour, refusal to consent for caesarean section, and service-related factors such as lack of transportation, and lack of equipment or expertise in the camp health facilities.

Fear of caesarean sections among Somali populations is a common finding in the literature [22–25] with the procedure being widely viewed as harming the woman and limiting her from having additional children. Moreover, caesareans may be seen as the *cause* of maternal-child death and disability, not the *prevention* of death and disability [24]. Such fear of death and disability from caesarean section may have developed from the real risks of surgery in under-resourced and unhygienic health facilities that have characterized services in Somalia in the past [26], as well as the increased risks faced by women with vertical uterine scars during subsequent pregnancies. The fear of reduced fecundity and the resultant loss of social status due to caesarean section are similarly valid fears within a society that places such a high value on childbearing [27, 28]. The serious social consequences for the woman, including social stigma, potential divorce, or re-negotiation of the dowry, results in a life-threateningly prolonged consent procedure where numerous elders must be consulted for approval.

These profound underlying fears may also contribute to the mistrust of health providers recommending the procedure, as evidenced by statements that health workers were performing caesareans only to earn more money or to remove extra organs for sale. This mistrust may be further compounded by the lack of a shared language, culture, and religion between the refugee population and the majority of health providers in the Dadaab camps, dynamics documented both in our study and others [28]. Mistrust of providers and consequent refusal of maternal interventions by Somali refugees in the Global North has been seen in other studies to result in delays in seeking care, poor adherence to treatments, and avoidance of emergency interventions including induction of labour and caesareans [24, 29, 30]. Binder et al.’s “maternal migration effect”, which explains how mistrust has contributed to an on-going increased risk of adverse maternal outcomes in women from high-mortality settings despite migrating to settings with better health services, appears to apply to Dadaab as well [30].

Resolving the underlying social fears of caesarean section will likely not occur in the immediate future and will require significant changes in societal gender roles. However, some practical steps may still be useful. Discussion about the birth plan, education and reassurance around caesarean section, and advance identification of decision makers for consent procedures should be incorporated into antenatal care visits in order to minimize delays during obstetric emergencies. This is particularly important in high-risk pregnancies. As well, stigma-reducing work in the community as a whole, highlighting healthy families who have had the procedure, would be valuable to address some of these issues in the short-term. Improving women’s experiences with facility-based delivery is also important to improve trust in services.

### Improving experiences of care

*Experiences of care*, including effective communication, respect, dignity, and emotional support, are considered key dimensions of the World Health Organization’s (WHO’s) quality of care framework for maternal and newborn care, alongside the more technical *provision of care* [31–33]*.* Continuous social support during childbirth has been shown to have a significant impact on both maternal satisfaction as well as maternal and newborn outcomes, including shortening labour, reducing rates of caesarean section, and improving 5 min APGAR scores [34]. “Companion of choice” also features in WHO’s guidelines for quality maternity care [32]. In our study, the inability to have a birth companion during facility-based delivery was raised as a reason why women may prefer to deliver at home or arrive late in labour, and is a factor that should be taken into account to improve women-centred care. Among the Somali diaspora, the use of a Somali doula in labour support has been found to be a successful intervention in trials in the United States [35, 36]. In Dadaab, adopting new hospital policies that support the presence of a birth companion during labour and delivery may help address some of the barriers to acceptability of care. The presence of a trusted family member or companion who can advocate for the labouring woman may additionally help reduce incidents of perceived disrespectful or neglectful care by health workers, ultimately increasing facility-delivery rates. Careful planning to ensure privacy of other patients as well allowing adequate working space for midwives will be required, but is feasible with consultation from staff, management and patients.

### Targeting home-based care practices

We found that mothers and grandmothers in our study often take a pluralistic approach to newborn health seeking, using traditional and religious remedies for the newborn in conjunction with biomedical care seeking. This is similar to much of the existing literature that indicates Somali populations often seek care from several different systems of healing, simultaneously or sequentially, including obtaining religious treatments, using traditional medicines, employing medicines obtained through pharmacies, and consulting bio-medical health care providers [37–40]. Our findings suggest further health education on danger signs that necessitate urgent biomedical care-seeking is needed in Dadaab, particularly for serious conditions such as convulsions that may be attributed to spiritual causes. As well, reducing transportation barriers by expanding emergency transport between home and the health centre beyond the Mama taxi provided to labouring women, may encourage more timely care-seeing with the sick newborn.

Home-based care practices including infant feeding and umbilical care practices impact newborn health outcomes [5]. Past research has found that early and exclusive breastfeeding is not the traditional norm in Somali populations, instead non-exclusive breastfeeding is practiced throughout the first two years of life [27, 41] with colostrum often believed to be spoiled milk and therefore discarded [38]. The situation in Dadaab is notably different however. Nutritional survey results in the camps the year prior to our study found rates of early initiation of breastfeeding ranged between 80 and 100% and exclusive breastfeeding rates ranged between 12.8 to 51.4%, depending on the camp (UNHCR: Standardized Expanded Nutritional Survey, Dadaab 2015, unpublished). Other research in Dadaab has shown decreasing use of bottle feeding and increased duration of exclusive breastfeeding after arriving in the camps [28]. Our participants similarly described a general shift away from discarding colostrum and delayed initiation of breastfeeding following health promotion efforts in the camps. However, we found mixed feeding from the first hours of life continues to be common practice, and is often initiated by elder female family members shortly after birth. Beliefs related to feeding only from the right breast, which have also been described in previous research [42], were found to persist among some women in the community.

Despite examples of positive behaviour change, the persistence of potentially harmful newborn care practices such as use of herbal remedies, delayed care-seeking, mixed feeding, and application of foreign substances to the umbilical stump suggest that a more extensive social and behaviour change communication approach is needed. Strengthening the skills and knowledge of community health workers in maternal and newborn health and behaviour change communication, and putting into place a more structured program of home visits may help promote healthy home-based newborn care [43]. Such an enhanced community-based approach that also includes grandmothers and fathers in health education efforts would recognize the importance of extended family members in maternal and newborn health decision-making.

### Limitations

This study has a number of limitations. Within Kenya, we completed the baseline assessment only in the Dadaab camps; we did not include other camps or urban/non-camp refugees. Consequently, the transferability of these findings to other settings or populations may be limited. Although we conducted the FGDs in the Somali language, we recorded notes in English. This may have resulted in translation errors or misinterpretations. Although differences in findings may exist between different clans, and based on the length of time since displacement, we did not analyse the data at this level of detail. Due to time constraints, we did not use thematic saturation as the end point for data collection and instead conducted a predetermined number of FGDs based on expected saturation. We were also unable to engage in member-checking with the community although we received feedback on our recommendations from health and managerial staff. The method of recruitment using word of mouth through partner NGOs may have resulted in participants with particularly strong views (either positive or negative) on health or other camp services to participate. In addition, as the focus groups were organized by UNHCR, this may have influenced responses of participants. For example, traditional birth attendants who continue to conduct home deliveries may not have felt comfortable to state this given the well-known negative perceptions of the practice by service providers. The positionalities of our team members, coming from Western and biomedical backgrounds, have undoubtedly influenced our interpretation and reporting of the findings. However, using local community members to facilitate the focus groups, regular debriefings, and the review of results after each session helped to minimize these limitations and increase the credibility of the findings. Further, we were able to triangulate our findings, both between various project components and the published literature, which gives us confidence regarding their trustworthiness.

## Conclusion

Key actions throughout the reproductive life cycle (preconception, pregnancy, childbirth and postnatal) and the across the continuum of care (home, community, and health facility) are required to improve maternal and neonatal outcomes. The findings from this study highlight the role of specific sociocultural beliefs and practices, and perceptions of health services on maternal and newborn health outcomes, providing valuable information to inform targeted improvements in health service provision that are tailored to the local context.
